# Editorial for Special Issue “Electric Transport and Magnetic Properties in Nanomaterials and Thin Films”

**DOI:** 10.3390/nano12244382

**Published:** 2022-12-09

**Authors:** Carlo Barone, Sergio Pagano

**Affiliations:** 1 Dipartimento di Fisica “E.R. Caianiello”, Università degli Studi di Salerno, 84084 Fisciano, SA, Italy; 2 INFN Gruppo Collegato di Salerno, c/o Università degli Studi di Salerno, 84084 Fisciano, SA, Italy; 3 CNR-SPIN, c/o Università degli Studi di Salerno, 84084 Fisciano, SA, Italy

Several nanomaterials and thin films have recently attracted much attention due to their peculiar electric transport and magnetic properties, such as the so-called magnetoresistance effect and the interplay between spin, orbital, charge, and structural degrees of freedom. All these phenomena have been the subject of a great deal of research, in view of possible applications in spin electronics and magnetism. Another relevant and emerging field of research in recent years is sustainability. Within this area, renewable polymers realized in the form of thin films and nanostructures have gained great popularity and the study of the electric transport properties is necessary to evaluate their integration into electronic circuitry. Finally, quantum technologies are attracting a large amount of attention in view of practical applications on quantum computation and quantum communication. In this respect, by exploiting the quantum features of nanostructures and nanoengineered materials, novel quantum devices can be designed.

All these topics have been the core of our Special Issue, entitled “Electric Transport and Magnetic Properties in Nanomaterials and Thin Films”, which includes six research papers: five articles and one review.

Advances in oxide thin-film deposition processes, allowing atomic-scale thickness control, have fundamentally increased research pm the low-dimensional effects in oxide-based heterostructures. Starting from this experimental advancement, a detailed investigation of infinite-layer electron-doped cuprates is reported. In particular, Sr_1-x_La_x_CuO_2_ thin films and SrCuO_2_/Sr_0.9_La_0.1_CuO_2_/SrCuO_2_ trilayers have been fabricated to study their electrical transport properties as a function of the doping and thickness of the central Sr_0.9_La_0.1_CuO_2_ layer [1]. The obtained results, indicating the key role played by the central layer thickness in the transport mechanisms, lead to the possibility of tuning the superconducting critical temperature of these devices by varying the system dimensionality.

The electrical conduction of infinite-layer electron-doped cuprates is also not well-understood in the normal-state region. Therefore, Sr_1−x_La_x_CuO_2_ thin films have been investigated as a function of doping, at temperatures above the superconducting transition temperature [2]. Optimal-doped samples show a non-Fermi liquid behavior above the critical temperature, while the over-doped samples are characterized by the presence of anti-ferromagnetic spin fluctuations, which are identified as the dominant transport normal-state mechanism of electron-doped cuprates [2].

Within the topic of sustainability, environmentally friendly energy storage devices, fabricated by using functional materials obtained from completely renewable resources, have been studied from the electrochemical point of view [3]. Different charge storage mechanisms have been identified and have been correlated to aging degradation effects. As a final result, increased capacitance retention rates for the casein and carboxymethylcellulose-based devices of 120% and 140% after 1000 cycles were observed, respectively [3].

A focus on green technologies, especially dedicated to environmental monitoring, has been also reported with electrical characterizations of environmentally friendly temperature sensors [4]. A detailed model describing the charge carrier accumulation, the faradaic charge transfer, and diffusion processes within the devices under the current-controlled has been proposed. A temperature sensitivity of about ~19 mV/K, long-term stability, and power consumption in the range of microwatts, suitable for indoor applications, have been observed for these sensors [4].

Finally, multiferroic and magnetodielectric effects and ferromagnetic behavior have been studied in multiferroic Pr_2_FeAlO_6_ double perovskite [5] and in semiconducting Co-doped ZnO [6], respectively. In more detail, the magnetoelectric effect in polycrystalline Pr_2_FeAlO_6_ samples was observed from the measurements of magnetically induced dielectric response and polarization [5]. Moreover, the magnetic and magneto-optical properties of Co-doped ZnO thin films have been discussed by considering the significant improvements in the properties induced by post-growth irradiation with atomic hydrogen [6]. The reported results suggest new complex oxide candidates for room-temperature multiferroic applications.

## Data Availability

Data sharing not applicable.
